# Proteoglycans as regulators of cancer biomarker landscapes: implications for precision oncology

**DOI:** 10.37349/etat.2026.1002409

**Published:** 2026-10-27

**Authors:** Aikaterini Berdiaki, Ioanna Spyridaki, Aristidis Tsatsakis, George Tzanakakis, Dragana Nikitovic

**Affiliations:** Amsterdam UMC, Netherlands; ^1^Department of Histology-Embryology, Medical School, University of Crete, 70013 Heraklion, Greece; ^2^Health Sciences Department, Universidad Ecotec, Samborondón EC092302, Ecuador; ^3^Biomedical Science and Technology Park, Sechenov I.M., First State Medical University, 119991 Moscow, Russia; ^4^Toxicology Lab, Medical School, University of Crete, 70013 Heraklion, Greece

**Keywords:** proteoglycans, glycosaminoglycan chains, extracellular matrix, cancer biomarkers, tumor microenvironment, biomarker heterogeneity, receptor signaling, precision oncology

## Abstract

In this review, we discuss the dual role of proteoglycans (PGs) in cancer biomarker biology. In addition to serving as biomarkers, PGs regulate the extracellular availability, signaling competence, compartmentalization, and interpretation of numerous disease-associated molecules within the tumor microenvironment. By controlling ligand sequestration, stability, and presentation, PGs influence the biological significance of molecular readouts and signaling-associated signatures. We further examine how extracellular matrix (ECM) remodeling processes, including proteolytic shedding, glycan remodeling, and extracellular vesicle (EV) release, generate circulating molecular signatures that reflect dynamic interactions between tumor cells and their surrounding microenvironment. In parallel, PG-dependent regulation of receptor signaling influences downstream transcriptional and inflammatory response profiles, while regional differences in PG distribution contribute to the formation of distinct ECM niches associated with local variations in signaling activity and molecular expression patterns. Viewed as a whole, these observations indicate that the detectability and clinical interpretation of cancer-associated markers are strongly influenced by PG-dependent extracellular regulation. This framework provides a mechanistic basis for understanding variability in marker performance and supports the incorporation of ECM context into biomarker research. Greater diagnostic precision and improved patient stratification will likely depend on approaches that consider the regulatory roles of PGs within the tumor microenvironment.

## Graphical abstract

**Graphical abstract.**
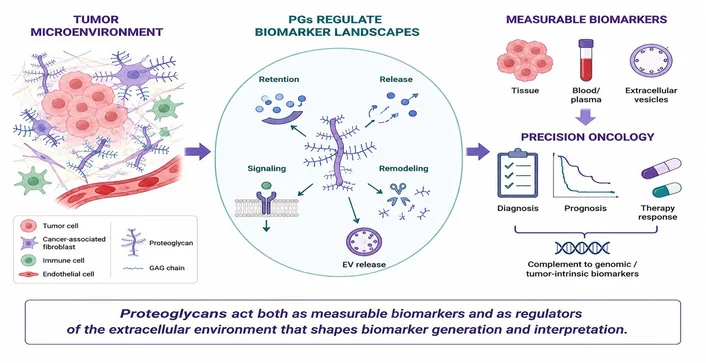
** PGs in cancer: regulators of biomarker landscapes and precision oncology.** PGs: proteoglycans; GAG: glycosaminoglycan; EV: extracellular vesicle.

## Introduction

Biomarker discovery and implementation play a crucial role in advancing precision oncology. Molecular markers are widely used for diagnosis, prognosis, patient stratification, and therapy monitoring. Despite significant progress, many of these markers fail to demonstrate consistent clinical performance across patient cohorts, tumor types, and disease stages. This variability is often attributed primarily to tumor genetic heterogeneity. Increasing evidence, however, indicates that non-genetic factors, particularly the tumor microenvironment, play a decisive and underappreciated role in shaping biomarker behavior [1, 2]. Recent advances in spatial omics further demonstrate that tumor-associated molecular profiles are strongly influenced by their spatial context and by interactions between malignant cells and surrounding stromal and immune compartments, highlighting the importance of tissue organization in biomarker interpretation [3].

The extracellular matrix (ECM) is a central component of healthy tissues and the tumor microenvironment, providing structural organization, as well as biochemical and biomechanical regulatory cues. Among ECM components, proteoglycans (PGs), consisting of core proteins decorated with glycosaminoglycan (GAG) chains, contribute to diverse aspects of tissue homeostasis and tumor biology. These molecules carry covalently attached GAG chains, such as heparan sulfate (HS), chondroitin sulfate, and dermatan sulfate, enabling interactions with a wide range of ligands, including growth factors, cytokines, morphogens, and extracellular enzymes [4, 5].

PGs comprise a structurally heterogeneous family of macromolecules whose classification reflects their localization and core protein organization. Cell-surface PGs include transmembrane syndecans and glycosylphosphatidylinositol (GPI)-anchored glypicans, which participate in ligand presentation and receptor signaling. Extracellular and pericellular matrix PGs comprise large aggregating molecules such as versican (VCAN) and aggrecan, involved in matrix organization and growth factor distribution. Basement membrane-associated PGs, including perlecan and agrin, regulate tissue architecture and signaling within specialized extracellular niches. In parallel, small leucine-rich PGs (SLRPs), such as decorin (DCN) and biglycan (BGN), represent a structurally distinct PG family characterized by leucine-rich repeat motifs within their core proteins that mediate interactions with receptors, collagen fibrils, and other matrix components. SLRPs are predominantly localized within interstitial ECM compartments, where they associate with collagen fibrils or remain soluble within the extracellular milieu. Functional diversity among PGs arises from variations in core protein structure, differences in GAG chain composition, and their ability to organize complex signaling networks within the tumor microenvironment [5, 6]. Importantly, as recently discussed, this diversity also influences the functional availability and signaling competence of biomarker-relevant molecules, supporting the emerging concept that PGs regulate biomarker landscapes [7].

PGs were initially investigated primarily as structural components of the ECM or as individual signaling regulators. More recently, however, they are recognized as active contributors to cancer progression, influencing proliferation, invasion, angiogenesis, immune modulation, and therapy resistance [8, 9]. Thus, many PGs, including syndecans, glypicans, perlecan, VCAN, and SLRPs, have been proposed as biomarkers in various cancers. Importantly, PGs occupy a unique position within biomarker biology. Unlike most biomarker classes, PGs can function both as measurable biomarkers and as active regulators of biomarker generation, distribution, and signaling activity. Consequently, changes in PG expression, structure, localization, or processing may influence not only the detection of PGs themselves but also the biological interpretation of numerous biomarker-relevant molecules operating within the same extracellular environment. Nevertheless, variability in expression, processing, localization, and detection continues to complicate their clinical implementation [10–14]. One limitation of current biomarker frameworks is that measured marker levels are often interpreted primarily as direct indicators of tumor-cell activity. Although this relationship may apply in certain settings, biomarker molecules function within extracellular environments that strongly affect their availability, localization, and signaling state. In this context, PGs, as dynamic components of the ECM, influence ligand sequestration, receptor engagement, and spatial organization within the tumor microenvironment [4, 15].

The following sections first examine PGs as biomarkers and then focus on their broader role in regulating biomarker landscapes. Particular emphasis is placed on ECM remodeling, receptor signaling, extracellular vesicle (EV) biology, and spatial heterogeneity, all of which influence biomarker generation, availability, and interpretation within tumors.

## PGs in cancer biomarker biology: from biomarker candidates to regulators of biomarker landscapes

PGs occupy a unique position within cancer biomarker biology because they function both as measurable molecular markers and as regulators of the extracellular processes that influence marker generation, availability, signaling competence, and interpretation. Several PG family members have attracted considerable interest as diagnostic, prognostic, and predictive biomarkers across multiple tumor types. Their expression in tissues, circulation, or EVs frequently reflects specific biological features of tumor progression, stromal remodeling, angiogenesis, immune regulation, and therapeutic response. Importantly, however, the level of clinical evidence supporting individual PGs differs considerably. Whereas glypican-3 (GPC3) in hepatocellular carcinoma (HCC) represents one of the more clinically advanced PG-associated biomarkers, most other PGs discussed below remain candidate or exploratory biomarkers whose diagnostic, prognostic, or predictive utility requires further validation [16].

Among the most extensively investigated PG biomarkers are the syndecans. Syndecans exist both as membrane-associated molecules and as soluble ectodomains generated through proteolytic shedding. In colorectal cancer, elevated circulating syndecan-1 (SDC1) levels have been associated with poor prognosis and enhanced epidermal growth factor receptor (EGFR) signaling [17–19]. Importantly, early studies identified elevated soluble SDC1 levels in patient circulation and linked them to disease progression and clinical outcome across multiple malignancies [20]. Higher pre-diagnostic serum SDC4 levels have recently been associated with increased breast cancer risk, particularly for hormone receptor-positive disease, supporting further investigation of circulating SDC4 as a potential early biomarker [18].

GPC3 represents one of the most established PG biomarkers in cancer. Widely studied in HCC, GPC3 has been evaluated in both tissue and serum specimens, often in combination with alpha-fetoprotein (AFP). Recent evidence further supports the translational relevance of GPC3 in HCC, with potential applications extending from diagnostic and prognostic assessment to patient stratification for emerging GPC3-targeted therapeutic approaches [16]. Although GPC3 remains one of the most extensively investigated HCC-associated markers, its diagnostic performance varies among patient cohorts [21–23]. This variability likely reflects differences in tumor differentiation, membrane expression, extracellular processing, and underlying liver pathology.

Additional cell-surface PGs have also emerged as potential biomarkers of aggressive disease. Chondroitin sulfate proteoglycan 4 (CSPG4; also known as NG2) has attracted increasing attention as a surface-associated and EV-associated biomarker in melanoma, where CSPG4-positive tumor-derived EVs have been isolated from patient plasma and proposed as potential biomarkers of disease progression [24]. CSPG4 is enriched in aggressive tumor cell populations and has been associated with invasive behavior and resistance to therapy, supporting its potential utility for tumor stratification and illustrating the biological heterogeneity that may contribute to biomarker variability [25].

Several members of the SLRP family have similarly been associated with tumor progression and clinical outcome. Clinical and experimental studies have linked reduced DCN expression with aggressive tumor behavior and loss of ECM-mediated tumor suppression [10, 26]. Elevated BGN expression has been associated with enhanced insulin-like growth factor I receptor (IGF-IR) signaling, tumor progression, and therapeutic resistance in osteosarcoma, supporting its potential value as a biomarker of aggressive disease [27]. In esophageal squamous cell carcinoma, cancer-associated fibroblast (CAF)-derived lumican accumulates within tumor stroma and has been significantly associated with poorer relapse-free and overall survival [28]. These observations indicate that alterations in SLRP expression may provide clinically relevant information regarding tumor behavior and microenvironmental remodeling.

Additional evidence further supports the utility of PGs as biomarkers across diverse cancer contexts. GPC3 remains one of the most extensively investigated biomarkers in HCC [16], where its expression is closely associated with activation of Wnt-dependent signaling pathways, although its diagnostic performance may vary among patient cohorts and disease stages [29, 30]. Likewise, stromal accumulation of hyalectan, VCAN, in desmoplastic and CAF-rich microenvironments has been linked to tumor progression, immune modulation, and adverse clinical outcomes [31]. Tumor-derived EVs carry heterogeneous molecular cargo capable of modifying stromal, immune, angiogenic, and ECM-remodeling processes, while PG-positive EV subpopulations, such as GPC1-positive circulating exosomes, have been identified in selected tumor settings [32]. In parallel, elevated BGN expression and the recent identification of exosomal BGN as a mediator of macrophage polarization and inflammatory signaling further support its relevance as both a biomarker and functional regulator of tumor progression [33]. Additional complexity arises from basement membrane-associated PGs such as perlecan, whose intact form and bioactive proteolytic fragments differentially regulate angiogenesis and extracellular signaling [34, 35]. Furthermore, circulating HS fragments generated during ECM remodeling have emerged as indicators of glycan turnover, inflammation, and heparanase activity within the tumor microenvironment [36, 37]. Importantly, neither the biological function nor the biomarker value of a given PG can be inferred from its abundance alone, as both depend on tumor type, cellular source, extracellular localization, GAG composition, proteolytic processing, and the surrounding signaling environment [5, 8, 13, 15, 38]. Figure 1 summarizes the principal classes of PGs being assessed as diagnostic, prognostic, and predictive biomarkers, as well as the biological sources through which they can be detected in tissues, circulation, and extracellular vesicles.

**Figure 1:**
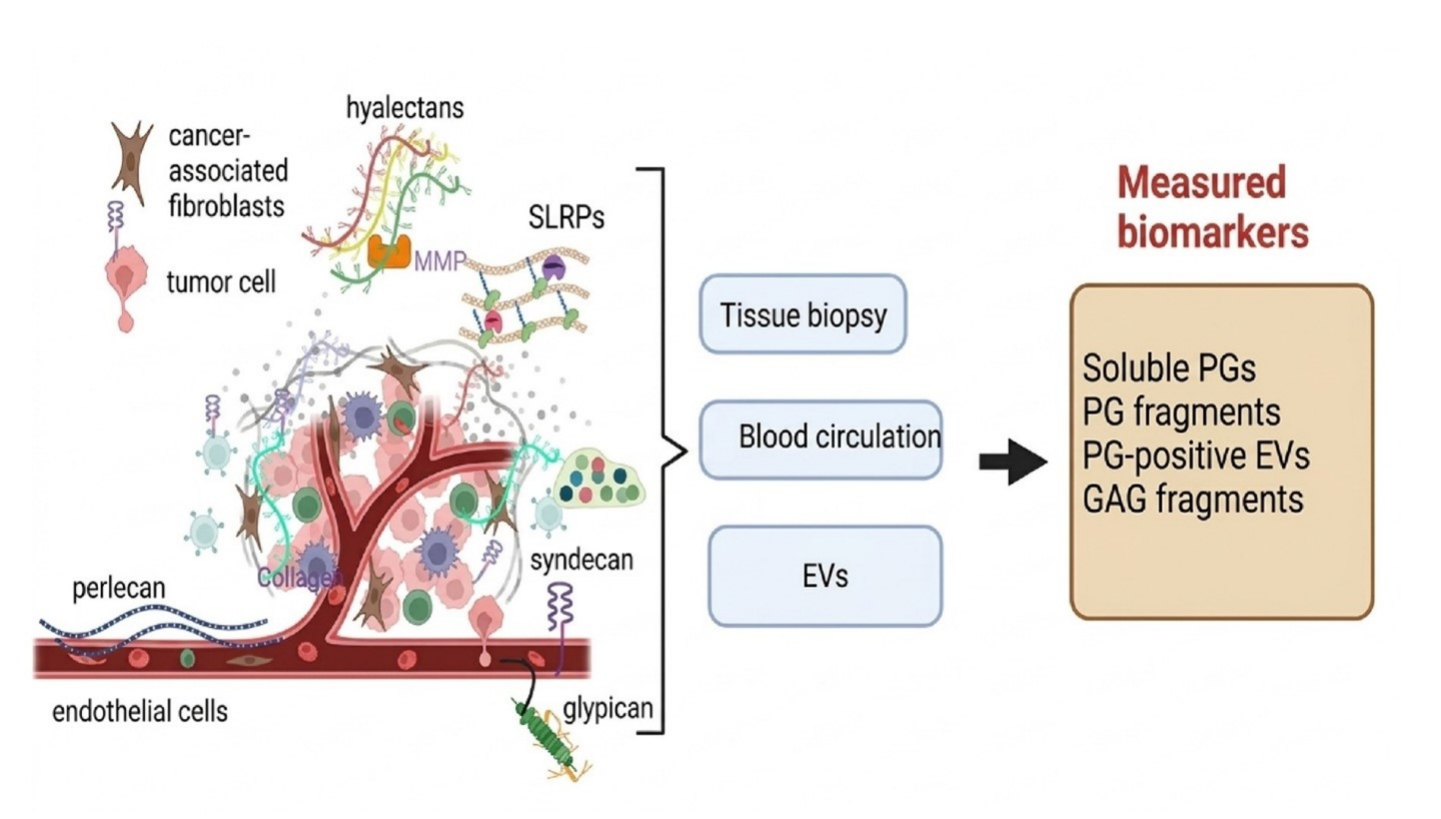
**PGs as cancer biomarkers: sources and clinical applications.** Cell-surface, extracellular, and basement membrane-associated PGs can be detected in tumor tissues, circulation, and EVs. Changes in their abundance, localization, shedding, and extracellular processing reflect tumor progression, stromal remodeling, angiogenesis, immune regulation, and therapeutic response. Consequently, PGs and their derived fragments are increasingly investigated as diagnostic, prognostic, and predictive biomarkers for precision oncology. SLRPs: small leucine-rich proteoglycans; PGs: proteoglycans; GAG: glycosaminoglycan; EVs: extracellular vesicles. Created in BioRender. Nikitovic, D. (2026) https://BioRender.com/80hz2sb.

Taken together, these examples illustrate the potential utility of PGs as biomarkers across diverse cancer types. However, the biological significance of PGs extends beyond their direct measurement. Many PGs additionally regulate extracellular signaling networks and influence the generation, availability, and interpretation of other classes of molecular markers within the tumor microenvironment. It should be emphasized, however, that direct clinical evidence linking PG-dependent extracellular regulation to altered diagnostic or predictive performance of established biomarkers remains limited. Thus, the concept of PG-regulated biomarker landscapes represents a mechanistically supported framework that integrates established functions of individual PGs in extracellular organization and signaling, but its broader clinical relevance requires prospective validation. Representative examples of PGs currently investigated as cancer biomarkers are summarized in Table 1.

**Table 1:** PGs and GAGs as biomarkers across cancer contexts.

**Molecule**	**Cancer context**	**Biomarker type**	**Key observation**	**Interpretation**	**References**
GPC3	HCC	Diagnostic/circulating	Variable serum-tissue correlation	Compartment-dependent detectability and tumor heterogeneity	[22, 29, 30]
SDC1	Multiple cancers, CRC	Circulating/prognostic	Elevated plasma levels linked to survival	Composite marker of tumor activity, ECM remodeling, and glycocalyx damage	[18–20]
SDC4	Breast cancer	Risk/early biomarker potential	Higher pre-diagnostic serum levels associated with subsequent breast cancer risk	Potential circulating biomarker requiring further validation	[18]
VCAN	Stromal-rich tumors	Stromal/prognostic	Accumulates in CAF-rich regions	Marker of desmoplasia and immune-modulating ECM	[11, 31]
CSPG4 (NG2)	Melanoma, HCC, TNBC	Surface/EV biomarker	Detected on tumor cells and EVs	Reflects invasive phenotype and tumor-ECM interactions	[39]
DCN	Multiple cancers	Stromal/prognostic	Reduced in aggressive tumors	Loss of tumor-suppressive ECM regulation	[10, 26]
BGN	Osteosarcoma, gastric cancer	Circulating/functional	Elevated and signaling-active	Reflects ECM remodeling and pathway activation	[27, 33]
Perlecan (HSPG2)	Angiogenic tumors	Matrix-associated	Intact vs. fragment dual roles	Distinguishes pro- vs. anti-angiogenic states	[34, 35]
PG-derived heparan sulfate fragments	Multiple cancers	Circulating/inflammatory	Released during ECM remodeling	Indicator of glycan turnover and inflammation	[36, 37]

GAGs: glycosaminoglycans; GPC3: glypican-3: SDC1: syndecan-1; CRC: colorectal carcinoma; ECM: extracellular matrix; VCAN: versican; CAF: cancer-associated fibroblast; CSPG4: chondroitin sulfate proteoglycan 4; HCC: hepatocellular carcinoma; TNBC: triple-negative breast cancer; DCN: decorin; BGN: biglycan; HSPG2: heparan sulfate proteoglycan 2.

Beyond their direct use as biomarkers, PGs influence the extracellular environment in which molecular markers are generated, distributed, and interpreted. Through coordinated contributions of their core proteins and GAG chains, PGs regulate ligand retention, extracellular compartmentalization, receptor accessibility, and molecular stability within tumors. These functions are particularly relevant in solid malignancies, where growth factors, cytokines, chemokines, and other signaling molecules are continuously retained, redistributed, or released within a highly dynamic ECM [9, 40, 41].

HSPGs, including syndecans, GPCs, perlecan, agrin, and collagen XVIII, provide some of the clearest examples of this regulatory role. HS chains bind numerous growth factors, morphogens, cytokines, chemokines, and extracellular enzymes through sulfation-dependent interactions. Alterations in HSPG expression, HS biosynthesis, and extracellular remodeling modify ligand distribution and signaling accessibility throughout the tumor microenvironment [15, 42].

The biological properties of HS are determined by chain length, domain organization, sulfation pattern, and epimerization. Variations in *N-,* 2-*O*-, 3-*O*-, and 6-*O*-sulfation generate structurally distinct domains that interact selectively with fibroblast growth factors (FGFs), vascular endothelial growth factor (VEGF), hepatocyte growth factor (HGF), platelet-derived growth factor (PDGF), Wnt ligands, chemokines, and extracellular proteases [5, 15]. Consequently, extracellular ligand availability does not necessarily correspond to signaling accessibility, as molecular activity remains strongly influenced by local matrix organization and PG-mediated presentation.

Perlecan illustrates how PGs influence biomarker interpretation through regulation of molecular compartmentalization. As a major basement membrane HSPG, perlecan contributes to tissue architecture while serving as a reservoir for growth factors including VEGF-A and FGF2 [34]. Changes in perlecan abundance, localization, or processing may therefore alter the extracellular distribution of signaling molecules and influence the biological significance of associated molecular readouts.

An additional layer of regulation is provided by enzymes that modify PG-associated GAG chains. Heparanase cleaves HS chains, altering ligand retention and facilitating the release of HS-associated growth factors, cytokines, and inflammatory mediators [37, 43]. Extracellular sulfatases such as sulfatase 2 (SULF2) further reshape HS sulfation patterns, modifying ligand accessibility and extracellular signaling landscapes [44, 45]. Through these mechanisms, GAG-remodeling enzymes influence the extracellular distribution and biological activity of numerous disease-associated molecules without necessarily changing their overall abundance. GAG metabolic and remodeling enzymes introduce an additional level of regulation into PG-associated biomarker biology. Heparanase-mediated cleavage of HS modifies extracellular ligand retention and promotes the release of HS-bound growth factors, cytokines, and inflammatory mediators, while extracellular sulfatases, particularly SULF1 and SULF2, selectively remodel HS sulfation domains and thereby modify ligand accessibility and receptor signaling [15, 37, 43–45]. Consequently, changes in the expression or activity of these enzymes may reflect functionally relevant remodeling of the tumor microenvironment that is not evident from measurement of PG core protein abundance alone. In this context, GAG-remodeling enzymes and the structural signatures they generate represent potential complementary biomarkers of extracellular signaling activity, although their clinical utility remains dependent on tumor context and requires further validation [46].

Available data indicate that PG-dependent regulation can affect molecular accessibility, extracellular compartmentalization, and ligand presentation throughout the tumor microenvironment. Measurements obtained from tissues or biological fluids therefore reflect not only cellular production but also matrix organization and extracellular remodeling processes. Consideration of these factors is essential for interpreting molecular signatures and understanding variability in marker performance across tumor types, patient cohorts, and disease stages [38, 40].

Beyond their direct use as biomarkers, PGs actively regulate the extracellular processes that influence biomarker generation, signaling competence, and molecular interpretation. As illustrated in Figure 2, PGs shape biomarker landscapes by controlling ligand sequestration, receptor organization, signaling pathway activation, transcriptional outputs, and ECM remodeling within the tumor microenvironment.

**Figure 2:**
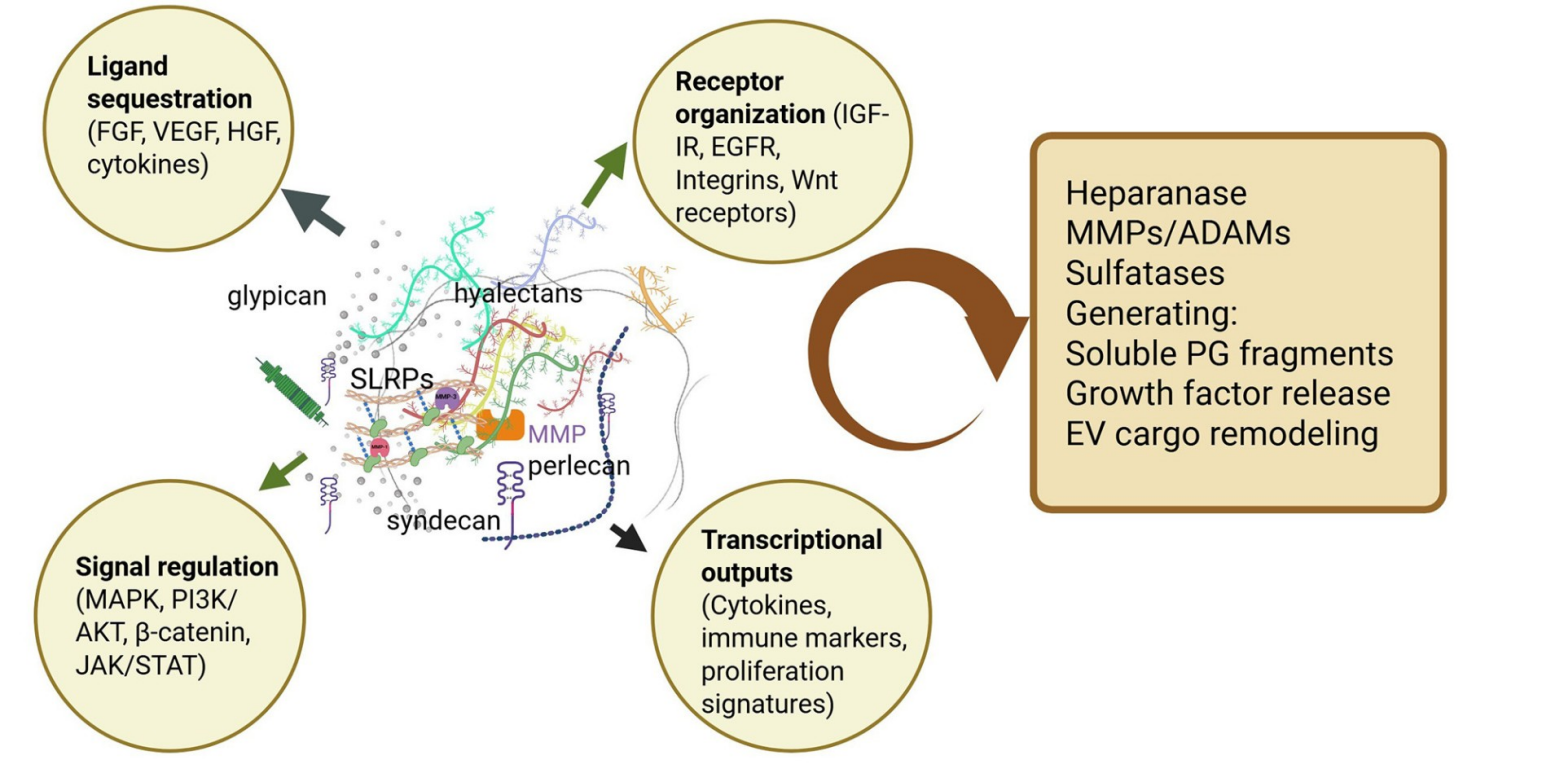
**PGs shape biomarker generation and signaling-associated molecular outputs.** PGs regulate the extracellular environment in which biomarkers are generated, distributed, and interpreted. Through coordinated actions of their core proteins and GAG chains, PGs influence ligand sequestration and presentation, receptor organization, signaling pathway activation, and downstream transcriptional responses. HSPGs, including syndecans, GPCs, and perlecan, bind and compartmentalize growth factors, cytokines, and morphogens, thereby controlling their bioavailability and signaling competence. In parallel, PGs facilitate the assembly of receptor signaling complexes involving receptor tyrosine kinases (RTKs), integrins, and Wnt receptors, modulating pathways such as MAPK, PI3K/AKT, β-catenin, and JAK/STAT. These events ultimately influence the expression of cytokines, immune-associated molecules, proliferation-related genes, and other biomarker-relevant transcriptional signatures. Continuous ECM remodeling by heparanase, MMPs, ADAM proteases, and extracellular sulfatases further reshapes these signaling landscapes through the generation of soluble PG fragments, growth factor release, and EV cargo remodeling. Consequently, biomarker measurements reflect not only cellular production but also PG-dependent extracellular regulation operating within the tumor microenvironment. The scheme represents major examples of PG-dependent regulatory mechanisms and is not intended to encompass the full diversity or context dependence of individual PG functions. GAG: glycosaminoglycan; FGF: fibroblast growth factor; VEGF: vascular endothelial growth factor; HGF: hepatocyte growth factor; MAPK: mitogen-activated protein kinase; PI3K: phosphoinositide 3-kinase; AKT: protein kinase B pathway; β-catenin: beta-catenin; JAK: Janus kinase; STAT: signal transducer and activator of transcription; SLRPs: small leucine-rich proteoglycans; IGF-IR: insulin-like growth factor I receptor; EGFR: epidermal growth factor receptor; MMPs: matrix metalloproteinases; ADAMs: a disintegrin and metalloproteinases; PG: proteoglycan; EV: extracellular vesicle. Created in BioRender. Nikitovic, D. (2026) https://BioRender.com/5dh3vmu.

## PG remodeling and the generation of circulating biomarker pools

ECM remodeling actively contributes to the generation of circulating biomarker pools. In many cases, biomarkers arise from coordinated interactions among tumor cells, stromal compartments, inflammatory cells, and matrix-remodeling enzymes operating within the tumor microenvironment [12, 40, 41]. Biomarkers detected in blood and other biofluids frequently originate from matrix remodeling, stromal activation, inflammatory responses, and vascular alterations occurring alongside tumor-cell release.

A major mechanism underlying this process is proteolytic remodeling of the ECM. Matrix metalloproteinases (MMPs), a disintegrin and metalloproteinase (ADAM) proteases, and ADAM with thrombospondin motifs (ADAMTS) family members cleave PG core proteins and associated matrix components, generating soluble ectodomains and biologically active fragments. Importantly, proteolytic processing may generate fragments that engage signaling pathways not activated by the intact parent molecule. ADAMTS-mediated cleavage of VCAN generates versikine, a bioactive matrikine implicated in the regulation of inflammatory and immune responses within the tumor microenvironment [11, 47]. Similarly, proteolytic processing of SLRPs can generate fragments with distinct inflammatory and signaling properties, linking ECM turnover with immune responses, inflammatory signaling, and pathways that influence biomarker profiles [12].

Proteolytic shedding of PG ectodomains represents an important source of circulating biomarker material. Protease-mediated cleavage releases soluble syndecan ectodomains carrying HS chains and associated ligands into the extracellular environment, thereby modifying local signaling and contributing to soluble PG pools [48, 49]. Elevated circulating SDC1 levels may reflect active stromal remodeling, endothelial glycocalyx damage, inflammatory activation, or tumor-associated protease activity in addition to tumor burden [18, 19].

Proteolytic remodeling additionally generates PG-derived fragments with signaling functions that differ markedly from those of intact molecules. Cleavage of perlecan produces endorepellin, a C-terminal fragment that exerts anti-angiogenic activity through VEGF receptor 2 (VEGFR2)- and integrin-associated mechanisms [35]. Circulating ECM fragments can therefore retain biological activity and continue to influence angiogenic, inflammatory, and stromal signaling pathways following their release.

EVs represent an additional source of circulating biomarker material, with PGs participating directly in EV biogenesis, cargo selection, and EV-mediated intercellular communication [50]. Syndecan–syntenin–ALIX complexes participate in exosome biogenesis [51]. Tumor-derived EVs carry heterogeneous molecular cargo capable of modifying stromal, immune, angiogenic, and ECM-remodeling processes, while PG-containing EV subpopulations have been identified in selected tumor settings [39]. Recent studies identified exosomal BGN released by gastric cancer cells as a mediator of macrophage polarization and chemokine (C-X-C motif) ligand 10 (CXCL10)/Janus kinase (JAK)/signal transducer and activator of transcription 1 pathway (STAT1) activation, demonstrating that PG-associated EV cargo can directly influence systemic inflammatory signaling and tumor progression [33]. EV cargo composition therefore reflects both tumor-cell intrinsic programs and ongoing ECM remodeling within the tumor microenvironment. In addition, EV-associated proteases and glycan-modifying enzymes may continue to modify extracellular signaling environments following vesicle release, thereby contributing to stromal activation, immune modulation, endothelial responses, and metastatic dissemination.

Remodeling of GAG chains further contributes to the release of biomarker-associated molecules into extracellular fluids. Heparanase-mediated cleavage of HS chains alters ligand retention and facilitates release of HS-associated growth factors, cytokines, and inflammatory mediators [37, 43]. Soluble HS fragments generated during this process can themselves function as inflammatory mediators capable of activating toll-like receptor (TLR)-dependent signaling pathways [36]. Extracellular sulfatases, including SULF2, further reshape HS sulfation patterns, with downstream effects on ligand accessibility, signaling activity, and therapeutic response [44, 45].

Circulating biomarker pools represent dynamic products of ECM turnover, proteolytic activity, EV trafficking, inflammatory signaling, and stromal-tumor interactions. PGs participate in each of these processes through their core proteins, associated GAG chains, and remodeling enzymes, linking ECM dynamics directly to the generation and biological interpretation of circulating biomarkers [38, 40].

## PG-dependent regulation of receptor signaling and biomarker outputs

Beyond regulating biomarker availability and release, PGs also influence how signaling pathways are activated, organized, and maintained within tumors. Many molecular signatures associated with receptor activation, inflammatory responses, or transcriptional programs reflect signaling activity that is strongly influenced by the surrounding extracellular environment. These effects arise through coordinated contributions of both PG core proteins and their associated GAG chains, which together regulate receptor organization, signaling intensity, pathway duration, and downstream transcriptional responses.

A major component of this regulation involves HSPGs, which function as co-receptors for multiple growth factor systems. HS chains stabilize interactions between ligands and their receptors, particularly within FGF, VEGF, and HGF signaling pathways, thereby influencing receptor activation thresholds and downstream signaling intensity [4, 52]. Under these conditions, ligand abundance alone provides limited information regarding signaling efficiency unless the local PG environment is also considered.

Cell-surface syndecans are particularly important in this context. Through both their extracellular and cytoplasmic domains, they interact with integrins, receptor tyrosine kinases (RTKs), and intracellular signaling proteins, forming multiprotein signaling platforms rather than isolated receptor pairs [14, 53]. SDC1 has been linked to coordinated IGF-IR and integrin signaling, affecting proliferative and migratory responses simultaneously [54, 55]. Such molecular organization generates compartmentalized signaling environments in which receptor activity depends on ligand concentration, receptor spatial arrangement, and membrane context.

GPC3 illustrates a distinct mechanism of signaling regulation. As a GPI-anchored HSPG, it localizes within membrane microdomains and modulates canonical Wnt/beta-catenin (β-catenin) signaling. In HCC, GPC3 enhances Wnt signaling by facilitating ligand-receptor interactions and promoting downstream transcriptional activation [29]. Changes in GPC3 expression therefore influence both pathway activity and downstream biomarker signatures associated with Wnt-dependent transcriptional programs.

Basement membrane PGs introduce an additional level of regulation. Perlecan (HSPG2), for example, supports angiogenic signaling through growth factor presentation, whereas its C-terminal fragment, endorepellin, suppresses angiogenic responses through VEGFR2-associated mechanisms [34, 35]. Different structural forms of the same PG may therefore correspond to distinct signaling states within the tumor microenvironment. Perlecan-associated signaling therefore varies according to its structural state, proteolytic processing, and extracellular localization.

SLRPs provide additional examples of context-dependent regulation of signaling networks. DCN interacts with several RTKs, including EGFR, mesenchymal-epithelial transition factor (MET), VEGFR2, and IGF-IR, thereby modulating downstream signaling in a receptor- and context-dependent manner [26, 56, 57]*.* DCN-mediated effects are strongly influenced by ECM composition, receptor availability, stromal organization, and inflammatory status. In breast carcinoma and glioblastoma, DCN suppresses EGFR- and MET-associated signaling and reduces tumor-cell proliferation, migration, and angiogenic responses [10, 26]. Recent work in HCC showed that *miR-181a* directly represses DCN, with downstream effects on proliferation- and apoptosis-related transcriptional programs, linking DCN loss to biomarker-relevant oncogenic signaling in liver cancer [58]. DCN additionally modulates receptor crosstalk, integrin organization, transforming growth factor beta (TGF-β) signaling, autophagy, endothelial-cell responses, and inflammatory pathways, thereby influencing multiple tumor-associated signaling networks [6, 59].

Distinct signaling consequences are also observed among SLRPs. In osteosarcoma, BGN regulates low-density lipoprotein receptor-related protein 6 (LRP6)/β-catenin/IGF-IR signaling and influences receptor activation, trafficking, and downstream proliferative responses [60, 61]. More recently, exosomal BGN released by gastric cancer cells was shown to promote M2 macrophage polarization and activate CXCL10-mediated JAK/STAT1 signaling [33]. These findings link PG-dependent extracellular regulation with receptor signaling, immune-cell behavior, and transcriptional programs associated with tumor progression.

PGs also influence receptor trafficking and signal duration. Syndecan-mediated internalization and recycling pathways affect receptor persistence at the plasma membrane and determine whether signaling remains transient or becomes sustained [62]. Differences in signal duration influence receptor fate, transcriptional responses, and downstream cellular behavior, introducing an additional source of variability in molecular readouts.

PGs function as integral components of signaling networks through effects on ligand presentation, receptor organization, trafficking, and signal persistence. Measurements based on receptor activation, transcriptional signatures, cytokine production, or pathway-associated molecular profiles require interpretation within the broader context of PG-dependent extracellular regulation, as measured expression levels do not necessarily correspond to ligand accessibility or signaling activity. The mechanistic roles of PGs are summarized in Table 2.

**Table 2:** Mechanistic roles of PGs in regulating signaling-dependent biomarker outputs.

**Mechanism**	**PG example**	**Molecular process**	**Biomarker consequence**	**Clinical implication**	**References**
Ligand sequestration	HSPGs (syndecans, perlecan)	HS-mediated ligand binding and presentation	Ligand abundance ≠ signaling activity	Limits interpretation of circulating growth factors and cytokines	[4, 15]
Co-receptor function	HSPGs	Stabilization of ligand-receptor interactions	Altered receptor activation thresholds	Explains variability in pathway-associated biomarkers	[4, 52]
Receptor organization	SDC1	Integrin-RTK signaling complexes	Compartmentalized signaling responses	Influences predictive biomarker performance	[14, 54]
Pathway activation	GPC3	Wnt/β-catenin signaling regulation	Distinct transcriptional biomarker signatures	Links PG expression to downstream signaling programs	[29]
RTK modulation	DCN	EGFR/MET/VEGFR2/IGF-IR interactions	Context-dependent signal attenuation and pathway rewiring	Reflects stromal control of tumor signaling	[26, 56, 57]
Oncogenic signaling	BGN	IGF-IR/LRP6/β-catenin and JAK/STAT activation	Enhanced proliferative and inflammatory signaling	Links ECM signaling to tumor progression and immune modulation	[27, 33, 60]
Receptor trafficking	Syndecans	Endocytosis and recycling pathways	Altered signal duration and transcriptional output	Affects biomarker kinetics and pathway persistence	[62]
Spatial compartmentalization	Multiple PGs	Formation of ECM signaling microdomains	Regional biomarker variability	Explains inconsistent biomarker performance across tumor regions	[1, 2]

HSPGs: heparan sulfate proteoglycans; HS: heparan sulfate; SDC1: syndecan-1; GPC3: glypican-3; β-catenin: beta-catenin; RTK: receptor tyrosine kinase; PG: proteoglycan; DCN: decorin; EGFR: epidermal growth factor receptor; MET: mesenchymal-epithelial transition factor; VEGFR2: vascular endothelial growth factor receptor 2; IGF-IR: insulin-like growth factor I receptor; BGN: biglycan; LRP6: low-density lipoprotein receptor-related protein 6; JAK: Janus kinase; STAT: signal transducer and activator of transcription; ECM: extracellular matrix.

## ECM-driven spatial heterogeneity and its impact on biomarker variability

A persistent challenge in biomarker research is the variability in biomarker performance across patients, tumor regions, and disease stages. While this variability is often attributed to genetic heterogeneity, emerging evidence indicates that ECM organization, particularly the spatial distribution and composition of PGs, contributes substantially to biomarker heterogeneity. Importantly, this heterogeneity does not arise randomly but reflects the formation of functionally distinct ECM niches that influence tumor behavior across malignancies including glioblastoma, HCC, and breast cancer [1, 2, 63]. Recent spatial proteomics analyses in glioblastoma identified discrete PG expression patterns involving CSPG4, CSPG5, protein tyrosine phosphatase receptor type Z1 (PTPRZ1), glypican family members, and TGF-β receptor III (betaglycan), which were associated with specific tumor-cell populations and microenvironmental states [64]. These observations indicate that local PG composition directly affects the functional identity of tumor niches, particularly in highly heterogeneous tumors such as glioblastoma.

Chondroitin sulfate PGs appear to be particularly important in establishing these regional microenvironments. CSPG4 (NG2), extensively studied in melanoma, triple-negative breast cancer (TNBC), and glioblastoma, is enriched in invasive and stem-like tumor-cell populations, especially at tumor-stroma interfaces. CSPG4 expression is preferentially associated with aggressive tumor phenotypes and has been linked to invasion and therapeutic resistance, supporting its potential value for identifying biologically distinct tumor cell populations [65]. The translational relevance of this compartmentalized expression is supported by recent CSPG4-targeting strategies in TNBC and glioblastoma models, where CSPG4 marks aggressive, therapeutically exploitable tumor populations [25, 66]. Similarly, the phosphacan-related PG PTPRZ1 defines glioma cell plasticity and localized signaling within specific tumor regions [67].

Basement membrane-associated PGs further contribute to spatial heterogeneity through regional processing within the ECM. Collagen XVIII, for example, undergoes cleavage generating endostatin, a potent anti-angiogenic fragment. In HCC and colorectal cancer, endostatin generation is associated with areas of active matrix remodeling, producing discrete anti-angiogenic microenvironments within otherwise highly angiogenic tumors [63, 68]. Consequently, angiogenesis-related biomarkers may reflect biologically divergent processes occurring simultaneously in different tumor regions.

Hyalectans, including VCAN and aggrecan, also contribute to ECM compartmentalization by regulating pericellular matrix architecture. In pancreatic ductal adenocarcinoma (PDAC) and breast cancer, VCAN-rich stromal regions are closely associated with CAF-dominated and immunosuppressive microenvironments [31, 69]. Additional evidence links VCAN patterns with tumor immune phenotype, indicating that VCAN may either support or restrict T-cell trafficking depending on its GAG context and stromal organization [70]. Aggrecan, although classically linked to cartilage biology, has additionally been implicated in gliomas and other brain tumors, where it creates a viscoelastic pericellular matrix permissive for tumor-cell motility and invasion [71, 72].

Additional transmembrane PGs help regulate localized signaling environments. Betaglycan (TGF-β receptor III), which exhibits heterogeneous expression in breast cancer, prostate cancer, and gliomas, modulates regional TGF-β signaling gradients. Since TGF-β signaling strongly influences epithelial-mesenchymal transition, fibrosis, and immune suppression, local differences in betaglycan expression are associated with region-specific biomarker signatures linked to these processes [73, 74].

Recent studies also support the concept that PG-rich ECM regions contribute to the formation of immune-exclusion niches. In pancreatic cancer, colorectal cancer, and glioblastoma, dense ECM regions enriched in specific PGs can restrict immune-cell infiltration while simultaneously sustaining immunosuppressive signaling pathways. Under such conditions, local immune activity may differ substantially from systemic immune biomarker profiles [75]. Recent breast cancer data further support this concept, showing that stromal VCAN accumulation can predict exclusion of CD8⁺ tumor-infiltrating lymphocytes, while VCAN proteolysis is associated with improved epithelial CD8⁺ T-cell infiltration, strengthening the view that PG accumulation and processing shape immune-related biomarker profiles [76]. Moreover, in a recent breast cancer analysis, it was further demonstrated that PG gene signatures may stratify tumor phenotypes and immune-related states, supporting the relevance of PG-defined spatial and molecular landscapes in clinical biomarker interpretation [77].

PG-dependent ECM organization also influences tissue mechanics, including stiffness, porosity, and matrix alignment. In breast cancer and HCC, these physical properties affect integrin-focal adhesion kinase (integrin-FAK) and yes-associated protein (YAP)/transcriptional coactivator with postsynaptic density protein 95-binding motif (TAZ) signaling pathways involved in proliferation, invasion, metabolic adaptation, and therapeutic resistance [1, 7, 63, 78]. Since these signaling pathways depend strongly on local ECM organization and mechanical tension, mechanotransduction-associated biomarkers frequently vary between tumor regions.

ECM spatial organization additionally affects therapeutic response. Distinct PG-defined tumor niches identified in glioblastoma, melanoma, and PDAC exhibit variable sensitivity to targeted therapies, immunotherapies, and anti-angiogenic agents [2]. As a result, neighboring tumor regions may respond differently to the same treatment, complicating interpretation of biomarkers measured in bulk tissue or circulation.

Overall, current evidence strongly supports the view that biomarker variability is closely associated with ECM spatial organization and the formation of region-specific PG-rich tumor niches. Spatial proteomics, multiplex imaging, and computationally integrated ECM analyses may therefore provide a more realistic representation of tumor organization and improve interpretation of biomarker datasets in precision oncology.

## Clinical implications and future perspectives

The mechanisms discussed above carry important implications for clinical biomarker interpretation. Biomarker measurements obtained from tissue or circulation frequently incorporate combined effects of tumor-cell activity, stromal remodeling, inflammatory signaling, endothelial alterations, and ECM turnover [79]. Taking these combined effects into account when interpreting molecular signature readouts, particularly relevant for circulating biomarkers and liquid biopsy approaches, could significantly improve clinical evaluation and therapeutic strategies [80]. From a clinical perspective, PGs occupy a dual position within biomarker biology. Several PG family members are themselves used as diagnostic, prognostic, or predictive markers, while PG-dependent extracellular regulation simultaneously influences the generation, detectability, and interpretation of numerous other biomarker classes.

Thus, although many PGs originate from stromal, endothelial, or inflammatory compartments rather than exclusively from malignant cells, this reflects the biological processes they are intended to report [8, 38, 69]. PG-associated biomarkers capture dynamic aspects of the tumor microenvironment, including ECM remodeling, angiogenesis, immune regulation, and stromal activation, that are not represented by tumor cell-intrinsic molecular alterations alone [8, 40, 69, 79]. Consequently, PGs are unlikely to replace genomic or mutation-based biomarkers used for molecular diagnosis and therapeutic stratification. Rather, their value lies in complementing these established biomarkers by providing information on the extracellular context that regulates tumor behavior, influences treatment response, and shapes the biological interpretation of molecular signatures [38, 40, 79].

A recent review specifically highlights soluble PGs and PG fragments in biological fluids as diagnostic, prognostic, or theragnostic circulating factors of pathological ECM remodeling, supporting their relevance for liquid biopsy strategies [38]. Elevated soluble SDC1 has been associated with disease burden and poor prognosis in multiple myeloma, where SDC1 also contributes to tumor vascularization, while increased serum SDC1 has been reported as a potential blood-based biomarker in PDAC [20, 81, 82]. Likewise, GPC1-positive EVs detected in patient plasma have been proposed as circulating markers of PDAC progression and metastatic dissemination, supporting the concept that PG-associated vesicular cargo captures tumor-specific signaling and stromal interactions [32]. Increased circulating hyaluronan fragments have additionally been associated with inflammatory matrix remodeling, vascular permeability, and poor clinical outcome in breast and colorectal cancers, indicating that ECM degradation products may reflect active stromal reprogramming rather than tumor burden alone [83–85].

PG biology additionally influences predictive markers and therapy stratification. Recent studies additionally support the use of PG-associated signatures for patient stratification, prediction of therapeutic response, and characterization of tumor immune states, particularly in breast cancer and HCC [58, 86]. PG-dependent regulation affects receptor signaling, mechanotransduction, stromal activation, angiogenesis, and immune-cell behavior, all of which are closely linked to tumor progression and therapeutic response. Emerging evidence further links PG expression patterns with tumor immune phenotypes and response to immunotherapy. Stromal VCAN accumulation, for example, has recently been associated with exclusion of CD8⁺ tumor-infiltrating lymphocytes, whereas VCAN proteolysis correlates with improved T-cell infiltration, suggesting that PG remodeling may generate clinically relevant immunological marker signatures [76]. Altered expression of specific PGs accompanies metastatic dissemination, immune evasion, and resistance to chemotherapy, targeted therapies, and immunotherapy in multiple cancers [71]. Noteworthy, in non-small cell lung cancer (NSCLC) patients treated with immune checkpoint inhibitors, anti-aggrecan and anti-PG autoantibodies increased after treatment in cases with higher programmed death-ligand 1 (PD-L1) expression, suggesting that PG-related immune reactivity may contribute to systemic immunological biomarker profiles [87]. Structural variability of GAG chains together with continuous ECM remodeling further modifies signaling dynamics and drug sensitivity across tumors and patient cohorts [88]. These parameters directly affect the predictive performance of current marker panels.

Regional matrix composition also influences the interpretation of clinical signaling readouts. Distinct tumor areas frequently differ in PG composition, stromal organization, immune-cell infiltration, vascularization, and mechanical properties. Biomarkers derived from bulk tissue analysis or circulation may therefore fail to capture biologically important tumor subpopulations. Spatial proteomics, multiplex imaging, digital pathology, and single-cell analyses increasingly allow characterization of ECM-associated heterogeneity and region-specific molecular signatures within tumors [3, 41].

The complexity of PG-dependent regulation further supports the development of integrative analytical strategies that combine proteomic, glycomic, transcriptomic, and spatial datasets. Computationally integrated ECM analyses may facilitate identification of context-dependent molecular signatures and improve interpretation of tumor-associated marker profiles. These approaches may additionally support development of multi-parametric diagnostic marker frameworks capable of reflecting tumor organization, stromal activity, and signaling dynamics more accurately than isolated molecular measurements.

PGs themselves are increasingly investigated as therapeutic targets and companion markers. Their involvement in growth factor signaling, immune modulation, angiogenesis, invasion, and matrix organization places them at central positions within tumor-associated signaling networks [89]. Indeed, recent breast cancer-focused analyses further support the role of PGs in prognosis, therapy response, metastatic niche formation, and modulation of the tumor immune landscape [86]. Enzymes that regulate GAG biosynthesis and remodeling, including heparanase and extracellular sulfatases, also directly participate in tumor progression and therapeutic resistance. Interventions targeting these pathways simultaneously influence tumor behavior, ECM organization, and biomarker profiles.

Importantly, PG biology is highly context-dependent. Distinct PG family members exhibit tissue-specific expression patterns, interact with different signaling networks, and participate in unique aspects of tumor biology. Consequently, their diagnostic, prognostic, or predictive utility cannot be generalized across malignancies. Instead, individual PGs should be evaluated within the biological context of specific tumor types, disease stages, and therapeutic settings. Such context dependency is fully consistent with the principles of precision oncology, which relies on tumor-specific biomarker combinations rather than universal molecular indicators. An important next step will be to determine which PG-dependent extracellular changes provide independent clinical information beyond established genomic, cellular, and circulating biomarkers, and in which tumor contexts such information can meaningfully improve patient stratification or therapeutic decision-making.

Several analytical and biological limitations currently restrict the clinical implementation of PG-based biomarkers. PG measurements may differ substantially according to whether the analyte is assessed in tissue, circulation, or EVs, while proteolytic processing and shedding can generate molecular forms that are not equivalent to the intact PG [12, 38, 51, 90]. Additional complexity arises from GAG structural heterogeneity, including differences in chain length, sulfation, epimerization, and extracellular remodeling, which are not captured by assays directed primarily against the core protein [15, 38, 43–45]. Furthermore, methodological differences in sample processing, detection platforms, and analytical thresholds complicate comparisons among studies. Standardization of analytical procedures, definition of the molecular species being measured, and prospective validation in tumor-specific patient cohorts will therefore be required before most PG-associated biomarkers can be incorporated into routine clinical practice [38].

Taken as a whole, current evidence indicates that ECM context is an integral component of marker biology and should be considered during molecular indicator discovery, validation, and clinical interpretation. Future biomarker strategies will likely require integrating molecular, spatial, and ECM-associated information to improve diagnostic precision, patient stratification, and therapeutic decision-making in precision oncology.

## Conclusions

PGs occupy a unique position within cancer biomarker biology. In addition to serving as measurable biomarkers, they influence the extracellular processes that determine molecular availability, compartmentalization, signaling accessibility, and biological interpretation. Through coordinated contributions of their core proteins, GAG chains, and associated remodeling enzymes, PGs participate in ligand retention, receptor organization, EV biology, matrix remodeling, and spatial regulation of signaling within tumors.

The evidence discussed throughout this review indicates that PG-dependent mechanisms may contribute to shaping molecular signatures relevant to cancer diagnosis, prognosis, and patient stratification. Consequently, variability in marker performance across patients, tumor types, and disease stages may reflect differences in extracellular organization and matrix remodeling in addition to tumor-intrinsic alterations.

Recent advances in spatial transcriptomics, multiplex imaging, liquid biopsy approaches, and systems-level computational analyses provide new opportunities to investigate PG-dependent regulation at increasing biological resolution. Integration of these approaches with functional studies of PGs and their associated signaling networks is expected to improve understanding of tumor heterogeneity and facilitate the identification of clinically relevant molecular signatures.

Current evidence suggests that PG-based biomarkers are likely to provide the greatest clinical value in malignancies characterized by pronounced ECM remodeling, desmoplastic reactions, active stromal signaling, or extensive immune-matrix interactions, including HCC, PDAC, glioblastoma, TNBC, melanoma, and osteosarcoma [7, 25, 27, 63, 66, 69, 71, 75, 76]. In these settings, PG-associated signatures may complement genomic and cellular biomarkers by capturing dynamic features of the tumor microenvironment that influence therapeutic response, immune regulation, and disease progression [1, 2, 7, 38, 40, 41, 63, 86]. Conversely, current evidence does not support the application of PGs as stand-alone biomarkers across all tumor entities [22, 23, 38, 40, 79, 88]. Their clinical implementation will likely require tumor-specific validation, standardized analytical methodologies, and integration with established molecular and cellular diagnostic platforms.

## Abbreviations

ADAM: a disintegrin and metalloproteinase
ADAMTS: a disintegrin and metalloproteinase with thrombospondin motifs
AFP: alpha-fetoprotein
AKT: protein kinase B pathway
BGN: biglycan
CAF: cancer-associated fibroblast
CSPG4 (NG2): chondroitin sulfate proteoglycan 4
CXCL10: chemokine (C-X-C motif) ligand 10
DCN: decorin
ECM: extracellular matrix
EGFR: epidermal growth factor receptor
EVs: extracellular vesicles
FGFs: fibroblast growth factors
GAG: glycosaminoglycan
GPC3: glypican-3
GPI: glycosylphosphatidylinositol
HCC: hepatocellular carcinoma
HGF: hepatocyte growth factor
HS: heparan sulfate
HSPGs: heparan sulfate proteoglycans
IGF-IR: insulin-like growth factor I receptor
integrin-FAK: integrin-focal adhesion kinase
JAK: Janus kinase
LRP6: low-density lipoprotein receptor-related protein 6
MAPK: mitogen-activated protein kinase
MET: mesenchymal-epithelial transition factor
MMPs: matrix metalloproteinases
NSCLC: non-small cell lung cancer
PDAC: pancreatic ductal adenocarcinoma
PD-L1: programmed death-ligand 1
PGs: proteoglycans
PI3K: phosphoinositide 3-kinase
PTPRZ1: protein tyrosine phosphatase receptor type Z1
RTKs: receptor tyrosine kinases
SDC1: syndecan-1
SLRPs: small leucine-rich proteoglycans
STAT1: signal transducer and activator of transcription 1 pathway
SULF2: sulfatase 2
TAZ: transcriptional coactivator with postsynaptic density protein 95-binding motif
TGF-β: transforming growth factor beta
TNBC: triple-negative breast cancer
VCAN: versican
VEGF: vascular endothelial growth factor
VEGFR2: vascular endothelial growth factor receptor 2
YAP: yes-associated protein
β-catenin: beta-catenin

## Declarations

### Copyright

© The Author(s) 2026.

## References

[1] Pickup MW, Mouw JK, Weaver VM (2014). The extracellular matrix modulates the hallmarks of cancer. EMBO Rep.

[2] Kai F, Laklai H, Weaver VM (2016). Force Matters: Biomechanical Regulation of Cell Invasion and Migration in Disease. Trends Cell Biol.

[3] Lee S, Kim G, Lee J, Lee AC, Kwon S (2024). Mapping cancer biology in space: applications and perspectives on spatial omics for oncology. Mol Cancer.

[4] Sarrazin S, Lamanna WC, Esko JD (2011). Heparan sulfate proteoglycans. Cold Spring Harb Perspect Biol.

[5] Iozzo RV, Schaefer L (2015). Proteoglycan form and function: A comprehensive nomenclature of proteoglycans. Matrix Biol.

[6] Theocharis AD, Skandalis SS, Gialeli C, Karamanos NK (2016). Extracellular matrix structure. Adv Drug Deliv Rev.

[7] Berdiaki A, Neagu M, Tzanakakis P, Spyridaki I, Pérez S, Nikitovic D (2024). Extracellular Matrix Components and Mechanosensing Pathways in Health and Disease. Biomolecules.

[8] Theocharis AD, Karamanos NK (2019). Proteoglycans remodeling in cancer: Underlying molecular mechanisms. Matrix Biol.

[9] Karamanos NK, Theocharis AD, Piperigkou Z, Manou D, Passi A, Skandalis SS (2021). A guide to the composition and functions of the extracellular matrix. FEBS J.

[10] Gubbiotti MA, Vallet SD, Ricard-Blum S, Iozzo RV (2016). Decorin interacting network: A comprehensive analysis of decorin-binding partners and their versatile functions. Matrix Biol.

[11] Timms KP, Maurice SB (2020). Context-dependent bioactivity of versican fragments. Glycobiology.

[12] Konstantaraki M, Thrapsanioti LN, Berdiaki A, Spyridaki I, Nikitovic D (2026). Interactions between small leucine‐rich proteoglycans and proteases: implications for tissue homeostasis and pathology. FEBS J.

[13] Giatagana EM, Berdiaki A, Tsatsakis A, Tzanakakis GN, Nikitovic D (2021). Lumican in Carcinogenesis—Revisited. Biomolecules.

[14] Afratis NA, Nikitovic D, Multhaupt HA, Theocharis AD, Couchman JR, Karamanos NK (2017). Syndecans - key regulators of cell signaling and biological functions. FEBS J.

[15] Marques C, Reis CA, Vivès RR, Magalhães A (2021). Heparan Sulfate Biosynthesis and Sulfation Profiles as Modulators of Cancer Signalling and Progression. Front Oncol.

[16] Tehrani HA, Zangi M, Fathi M, Vakili K, Hassan M, Rismani E (2025). GPC-3 in hepatocellular carcinoma; A novel biomarker and molecular target. Exp Cell Res.

[17] Wang H, Jin H, Beauvais DM, Rapraeger AC (2014). Cytoplasmic Domain Interactions of Syndecan-1 and Syndecan-4 with α6β4 Integrin Mediate Human Epidermal Growth Factor Receptor (HER1 and HER2)-dependent Motility and Survival. J Biol Chem.

[18] Gabrielsen E, Wilsgaard T, Frydenberg H, Lofterød T, Dalen SM, Mortensen E (2025). Higher pre-diagnostic serum syndecan-4 levels are associated with increased breast cancer risk: a case-cohort study. Breast Cancer Res Treat.

[19] Tanaka H, Hayashi H, Tomita H, Tokumaru Y, Fukada M, Tajima JY (2024). Association of Preoperative and Postoperative Plasma Syndecan-1 and Colorectal Cancer Outcome. Anticancer Res.

[20] Seidel C, Sundan A, Hjorth M, Turesson I, Dahl IMS, Abildgaard N (2000). Serum syndecan-1: a new independent prognostic marker in multiple myeloma. Blood.

[21] El-Saadany S, El-Demerdash T, Helmy A, Mayah WW, El-Sayed Hussein B, Hassanien M (2018). Diagnostic Value of Glypican-3 for Hepatocellular Carcinomas. Asian Pac J Cancer Prev.

[22] Batbaatar B, Gurbadam U, Tuvshinsaikhan O, Narmandakh NE, Khatanbaatar G, Radnaabazar M (2024). Evaluation of glypican3 in patients with hepatocellular carcinoma. Mol Clin Oncol.

[23] Lembas A, Mikuła T, Suchacz M, Kozłowska J, Barczak S, Badura B (2024). Normal Glypican-3 Serum Levels Do Not Eliminate the Risk of Hepatocellular Carcinoma. GALICIAN MED J.

[24] Sharma P, Ludwig S, Muller L, Hong CS, Kirkwood JM, Ferrone S (2018). Immunoaffinity‐based isolation of melanoma cell‐derived exosomes from plasma of patients with melanoma. J Extracell Vesicles.

[25] Mungra N, Biteghe FAN, Malindi Z, Huysamen AM, Karaan M, Hardcastle NS (2023). CSPG4 as a target for the specific killing of triple-negative breast cancer cells by a recombinant SNAP-tag-based antibody-auristatin F drug conjugate. J Cancer Res Clin Oncol.

[26] Iozzo RV, Buraschi S, Genua M, Xu SQ, Solomides CC, Peiper SC (2011). Decorin Antagonizes IGF Receptor I (IGF-IR) Function by Interfering with IGF-IR Activity and Attenuating Downstream Signaling. J Biol Chem.

[27] Giatagana EM, Berdiaki A, Gaardløs M, Samsonov SA, Tzanakakis GN, Nikitovic D (2022). Biglycan Interacts with Type I Insulin-like Receptor (IGF-IR) Signaling Pathway to Regulate Osteosarcoma Cell Growth and Response to Chemotherapy. Cancers (Basel).

[28] Yamauchi N, Kanke Y, Saito K, Okayama H, Yamada S, Nakajima S (2021). Stromal expression of cancerassociated fibroblastrelated molecules, versican and lumican, is strongly associated with worse relapsefree and overall survival times in patients with esophageal squamous cell carcinoma. Oncol Lett.

[29] Capurro MI, Xiang YY, Lobe C, Filmus J (2005). Glypican-3 Promotes the Growth of Hepatocellular Carcinoma by Stimulating Canonical Wnt Signaling. Cancer Res.

[30] Filmus J, Capurro M, Rast J (2008). Glypicans. Genome Biol.

[31] Pappas AG, Magkouta S, Pateras IS, Skianis I, Moschos C, Vazakidou ME (2018). Versican modulates tumor-associated macrophage properties to stimulate mesothelioma growth. OncoImmunology.

[32] Melo SA, Luecke LB, Kahlert C, Fernandez AF, Gammon ST, Kaye J (2015). Glypican-1 identifies cancer exosomes and detects early pancreatic cancer. Nature.

[33] Li W, Wei H, Liu J, Zhao Z, Wang F, Qiao L (2025). Exosomal Biglycan promotes gastric cancer progression via M2 polarization and CXCL10-mediated JAK/STAT1 activation. Cancer Lett.

[34] Zoeller JJ, Whitelock JM, Iozzo RV (2009). Perlecan regulates developmental angiogenesis by modulating the VEGF-VEGFR2 axis. Matrix Biol.

[35] Goyal A, Pal N, Concannon M, Paul M, Doran M, Poluzzi C (2011). Endorepellin, the angiostatic module of perlecan, interacts with both the α2β1 integrin and vascular endothelial growth factor receptor 2 (VEGFR2): a dual receptor antagonism. J Biol Chem.

[36] Goodall KJ, Poon IK, Phipps S, Hulett MD (2014). Soluble Heparan Sulfate Fragments Generated by Heparanase Trigger the Release of Pro-Inflammatory Cytokines through TLR-4. PLoS ONE.

[37] Vlodavsky I, Singh P, Boyango I, Gutter-Kapon L, Elkin M, Sanderson RD (2016). Heparanase: From basic research to therapeutic applications in cancer and inflammation. Drug Resist Updat.

[38] Kemberi M, Minns AF, Santamaria S (2024). Soluble Proteoglycans and Proteoglycan Fragments as Biomarkers of Pathological Extracellular Matrix Remodeling. Proteoglycan Res.

[39] Sharma P, Diergaarde B, Ferrone S, Kirkwood JM, Whiteside TL (2020). Melanoma cell-derived exosomes in plasma of melanoma patients suppress functions of immune effector cells. Sci Rep.

[40] Petersen EV, Chudakova DA, Skorova EY, Anikin V, Reshetov IV, Mynbaev OA (2020). The Extracellular Matrix-Derived Biomarkers for Diagnosis, Prognosis, and Personalized Therapy of Malignant Tumors. Front Oncol.

[41] Huang J, Zhang L, Wan D, Zhou L, Zheng S, Lin S (2021). Extracellular matrix and its therapeutic potential for cancer treatment. Signal Transduct Target Ther.

[42] De Pasquale V, Pavone LM (2020). Heparan Sulfate Proteoglycan Signaling in Tumor Microenvironment. Int J Mol Sci.

[43] Arvatz G, Weissmann M, Ilan N, Vlodavsky I (2016). Heparanase and cancer progression: New directions, new promises. Hum Vaccin Immunother.

[44] Lui NS, Yang YW, van Zante A, Buchanan P, Jablons DM, Lemjabbar-Alaoui H (2016). SULF2 Expression Is a Potential Diagnostic and Prognostic Marker in Lung Cancer. PLOS ONE.

[45] Zaki MYW, Alhasan SF, Shukla R, McCain M, Laszczewska M, Geh D (2022). Sulfatase-2 from Cancer Associated Fibroblasts: An Environmental Target for Hepatocellular Carcinoma?. Liver Cancer.

[46] Han M, Zhu H, Chen X, Luo X (2024). 6-O-endosulfatases in tumor metastasis: heparan sulfate proteoglycans modification and potential therapeutic targets. Am J Cancer Res.

[47] Hope C, Foulcer S, Jagodinsky J, Chen SX, Jensen JL, Patel S (2016). Immunoregulatory roles of versican proteolysis in the myeloma microenvironment. Blood.

[48] Manon-Jensen T, Multhaupt HA, Couchman JR (2013). Mapping of matrix metalloproteinase cleavage sites on syndecan‐1 and syndecan‐4 ectodomains. FEBS J.

[49] Ramani VC, Purushothaman A, Stewart MD, Thompson CA, Vlodavsky I, Au JL (2013). The heparanase/syndecan‐1 axis in cancer: mechanisms and therapies. FEBS J.

[50] Liao H, Zhang C, Wang F, Jin F, Zhao Q, Wang X (2024). Tumor-derived extracellular vesicle proteins as new biomarkers and targets in precision oncology. J Mol Med.

[51] Baietti MF, Zhang Z, Mortier E, Melchior A, Degeest G, Geeraerts A (2012). Syndecan–syntenin–ALIX regulates the biogenesis of exosomes. Nat Cell Biol.

[52] Ornitz DM, Itoh N (2015). The Fibroblast Growth Factor signaling pathway. Wiley Interdiscip Rev Dev Biol.

[53] Couchman JR (2010). Transmembrane Signaling Proteoglycans. Annu Rev Cell Dev Biol.

[54] Beauvais DM, Rapraeger AC (2010). Syndecan-1 couples the insulin-like growth factor-1 receptor to inside-out integrin activation. J Cell Sci.

[55] Rapraeger AC (2013). Synstatin: a selective inhibitor of the syndecan-1-coupled IGF1R-αvβ3 integrin complex in tumorigenesis and angiogenesis. FEBS J.

[56] Goldoni S, Humphries A, Nyström A, Sattar S, Owens RT, McQuillan DJ (2009). Decorin is a novel antagonistic ligand of the Met receptor. J Cell Biol.

[57] Iozzo RV, Moscatello DK, McQuillan DJ, Eichstetter I (1999). Decorin Is a Biological Ligand for the Epidermal Growth Factor Receptor. J Biol Chem.

[58] Assal RA, Abd El-Bary RBE, Youness RA, Abdelrahman MM, Zahran H, Hosny KA (2024). OncomiR-181a promotes carcinogenesis by repressing the extracellular matrix proteoglycan decorin in hepatocellular carcinoma. BMC Gastroenterol.

[59] Neill T, Schaefer L, Iozzo RV (2016). Decorin as a multivalent therapeutic agent against cancer. Adv Drug Deliv Rev.

[60] Aggelidakis J, Berdiaki A, Nikitovic D, Papoutsidakis A, Papachristou DJ, Tsatsakis AM (2018). Biglycan Regulates MG63 Osteosarcoma Cell Growth Through a LPR6/β-Catenin/IGFR-IR Signaling Axis. Front Oncol.

[61] Giatagana EM, Berdiaki A, Gaardløs M, Tsatsakis AM, Samsonov SA, Nikitovic D (2022). Rapamycin-induced autophagy in osteosarcoma cells is mediated via the biglycan/Wnt/β-catenin signaling axis. Am J Physiol-Cell Physiol.

[62] Le Roy C, Wrana JL (2005). Signaling and Endocytosis: A Team Effort for Cell Migration. Dev Cell.

[63] Boaru DL, De Leon-Oliva D, De Castro-Martinez P, Garcia-Montero C, Fraile-Martinez O, García-González B (2025). Extracellular matrix dysregulation in aging, calcification, and cancer diseases: insights into cellular senescence, inflammation, and novel therapeutic strategies. Int J Biol Sci.

[64] Day ZI, Roberts-Thomson S, Nouri YJ, Dalton NS, Wang SS, Davenport A (2025). Defining the extracellular matrix for targeted immunotherapy in adult and pediatric brain cancer. NPJ Precis Oncol.

[65] Campoli M, Ferrone S, Wang X (2010). Functional and Clinical Relevance of Chondroitin Sulfate Proteoglycan 4. Adv Cancer Res.

[66] Xiong Q, Yin B, Jiang H, Qiu Y, Shi G, Xu J (2025). Targeting CSPG4 enhances the anti-tumor activity of CAR-NK cells for glioblastoma. Cell Oncol (Dordr).

[67] Fujikawa A, Sugawara H, Tanaka T, Matsumoto M, Kuboyama K, Suzuki R (2017). Targeting PTPRZ inhibits stem cell-like properties and tumorigenicity in glioblastoma cells. Sci Rep.

[68] O'Reilly MS, Boehm T, Shing Y, Fukai N, Vasios G, Lane WS (1997). Endostatin: An Endogenous Inhibitor of Angiogenesis and Tumor Growth. Cell.

[69] Wight TN, Kang I, Evanko SP, Harten IA, Chang MY, Pearce OMT (2020). Versican—A Critical Extracellular Matrix Regulator of Immunity and Inflammation. Front Immunol.

[70] Hirani P, McDermott J, Rajeeve V, Cutillas PR, Jones JL, Pennington DJ (2024). Versican Associates with Tumor Immune Phenotype and Limits T-cell Trafficking via Chondroitin Sulfate. Cancer Res Commun.

[71] Samaržija I (2025). Proteoglycans in Prostate Cancer Progression and Therapy Resistance. Medicina (Kaunas).

[72] Zimmermann DR, Dours-Zimmermann MT (2008). Extracellular matrix of the central nervous system: from neglect to challenge. Histochem Cell Biol.

[73] Hempel N, How T, Dong M, Murphy SK, Fields TA, Blobe GC (2007). Loss of Betaglycan Expression in Ovarian Cancer: Role in Motility and Invasion. Cancer Res.

[74] Gordon KJ, Dong M, Chislock EM, Fields TA, Blobe GC (2008). Loss of type III transforming growth factor beta receptor expression increases motility and invasiveness associated with epithelial to mesenchymal transition during pancreatic cancer progression. Carcinogenesis.

[75] Maletin N (2026). From immune exclusion to exhaustion: tumor microenvironment drives therapy response. Front Immunol.

[76] Emmerich PB, Qyli T, Johnson KA, Chaudhuri S, Clark KM, Verhagen NB (2025). Stromal Versican Accumulation and Proteolysis Regulate the Infiltration of CD8+ T Cells in Breast Cancer. Cancers (Basel).

[77] Buraschi S, Pascal G, Liberatore F, Iozzo RV (2025). Comprehensive investigation of proteoglycan gene expression in breast cancer: Discovery of a unique proteoglycan gene signature linked to the malignant phenotype. Proteoglycan Res.

[78] Dupont S, Morsut L, Aragona M, Enzo E, Giulitti S, Cordenonsi M (2011). Role of YAP/TAZ in mechanotransduction. Nature.

[79] Purkayastha K, Dhar R, Pethusamy K, Srivastava T, Shankar A, Rath GK (2023). The issues and challenges with cancer biomarkers. J Cancer Res Ther.

[80] Ma L, Guo H, Zhao Y, Liu Z, Wang C, Bu J (2024). Liquid biopsy in cancer: current status, challenges and future prospects. Signal Transduct Target Ther.

[81] Yablecovitch D, Ben-Horin S, Picard O, Yavzori M, Fudim E, Nadler M (2022). Serum Syndecan-1: A Novel Biomarker for Pancreatic Ductal Adenocarcinoma. Clin Transl Gastroenterol.

[82] Purushothaman A, Uyama T, Kobayashi F, Yamada S, Sugahara K, Rapraeger AC (2010). Heparanase-enhanced shedding of syndecan-1 by myeloma cells promotes endothelial invasion and angiogenesis. Blood.

[83] Auvinen P, Tammi R, Parkkinen J, Tammi M, Agren U, Johansson R (2000). Hyaluronan in Peritumoral Stroma and Malignant Cells Associates with Breast Cancer Spreading and Predicts Survival. Am J Pathol.

[84] Tammi RH, Kultti A, Kosma VM, Pirinen R, Auvinen P, Tammi MI (2008). Hyaluronan in human tumors: Pathobiological and prognostic messages from cell-associated and stromal hyaluronan. Semin Cancer Biol.

[85] Bouga H, Tsouros I, Bounias D, Kyriakopoulou D, Stavropoulos MS, Papageorgakopoulou N (2010). Involvement of hyaluronidases in colorectal cancer. BMC Cancer.

[86] Vigo-Díaz N, López-Cortés R, Velo-Heleno I, Rodríguez-Silva L, Núñez C (2025). Proteoglycans in Breast Cancer: Friends and Foes. Biomolecules.

[87] Gwin ME, von Itzstein MS, Liu J, Fattah FJ, Mu-Mosley H, SoRelle JA (2025). Association of PD-L1 expression with systemic immune parameters in non-small cell lung cancer. Transl Lung Cancer Res.

[88] Depau L, Brunetti J, Falciani C, Mandarini E, Zanchi M, Paolocci MF (2025). Targeting heparan sulfate proteoglycans as an effective strategy for inhibiting cancer cell migration and invasiveness compared to heparin. Front Cell Dev Biol.

[89] Dituri F, Gigante G, Scialpi R, Mancarella S, Fabregat I, Giannelli G (2022). Proteoglycans in Cancer: Friends or Enemies? A Special Focus on Hepatocellular Carcinoma. Cancers (Basel).

[90] Kalluri R, LeBleu VS (2020). The biology, function, and biomedical applications of exosomes. Science.

